# First-in-human clinical series of a novel conformable large-lattice pulsed field ablation catheter for pulmonary vein isolation

**DOI:** 10.1093/europace/euae090

**Published:** 2024-04-08

**Authors:** Vivek Y Reddy, Elad Anter, Petr Peichl, Gediminas Rackauskas, Jan Petru, Moritoshi Funasako, Jacob S Koruth, Germanas Marinskis, Mohit Turagam, Audrius Aidietis, Josef Kautzner, Andrea Natale, Petr Neuzil

**Affiliations:** Department of Cardiology, Icahn School of Medicine at Mount Sinai, One Gustave Levy Place, Box 1030, New York, NY, USA; Department of Cardiology, Homolka Hospital, Prague, Czech Republic; Division of Cardiovascular Medicine, Shamir Medical Center, Be'er Yaakov, Tel Aviv, Israel; Department of Cardiology, Institute for Clinical and Experimental Medicine-IKEM, Prague, Czech Republic; Department of Cardiology, Vilnius University, Vilnius, Lithuania; Department of Cardiology, Homolka Hospital, Prague, Czech Republic; Department of Cardiology, Homolka Hospital, Prague, Czech Republic; Department of Cardiology, Icahn School of Medicine at Mount Sinai, One Gustave Levy Place, Box 1030, New York, NY, USA; Department of Cardiology, Vilnius University, Vilnius, Lithuania; Department of Cardiology, Icahn School of Medicine at Mount Sinai, One Gustave Levy Place, Box 1030, New York, NY, USA; Department of Cardiology, Vilnius University, Vilnius, Lithuania; Department of Cardiology, Institute for Clinical and Experimental Medicine-IKEM, Prague, Czech Republic; Texas Cardiac Arrhythmia Institute, St. David’s Medical Center, Austin, TX, USA; Department of Biomedicine and Prevention, Division of Cardiology, University of Tor Vergata, Rome, Italy; Department of Cardiology, Homolka Hospital, Prague, Czech Republic

**Keywords:** Atrial fibrillation, Single-shot, Catheter ablation, Pulsed field ablation, Lesion durability, Electroanatomical mapping system

## Abstract

**Aims:**

Pulsed field ablation (PFA) has significant advantages over conventional thermal ablation of atrial fibrillation (AF). This first-in-human, single-arm trial to treat paroxysmal AF (PAF) assessed the efficiency, safety, pulmonary vein isolation (PVI) durability and one-year clinical effectiveness of an 8 Fr, large-lattice, conformable single-shot PFA catheter together with a dedicated electroanatomical mapping system.

**Methods and results:**

After rendering the PV anatomy, the PFA catheter delivered monopolar, biphasic pulse trains (5–6 s per application; ∼4 applications per PV). Three waveforms were tested: PULSE1, PULSE2, and PULSE3. Follow-up included ECGs, Holters at 6 and 12 months, and symptomatic and scheduled transtelephonic monitoring. The primary and secondary efficacy endpoints were acute PVI and post-blanking atrial arrhythmia recurrence, respectively. Invasive remapping was conducted ∼75 days post-ablation. At three centres, PVI was performed by five operators in 85 patients using PULSE1 (*n* = 30), PULSE2 (*n* = 20), and PULSE3 (*n* = 35). Acute PVI was achieved in 100% of PVs using 3.9 ± 1.4 PFA applications per PV. Overall procedure, transpired ablation, PFA catheter dwell and fluoroscopy times were 56.5 ± 21.6, 10.0 ± 6.0, 19.1 ± 9.3, and 5.7 ± 3.9 min, respectively. No pre-defined primary safety events occurred. Upon remapping, PVI durability was 90% and 99% on a per-vein basis for the total and PULSE3 cohort, respectively. The Kaplan–Meier estimate of one-year freedom from atrial arrhythmias was 81.8% (95% CI 70.2–89.2%) for the total, and 100% (95% CI 80.6–100%) for the PULSE3 cohort.

**Conclusion:**

Pulmonary vein isolation (PVI) utilizing a conformable single-shot PFA catheter to treat PAF was efficient, safe, and effective, with durable lesions demonstrated upon remapping.

## Graphical Abstract

**Graphical Abstract:**
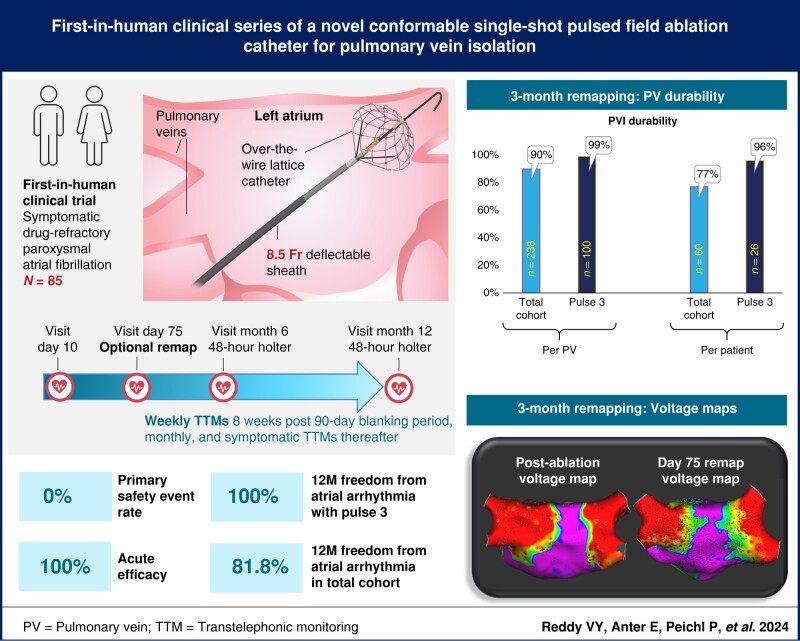

**What’s new?**

- In patients with paroxysmal atrial fibrillation, a novel conformable large-lattice pulsed field ablation (PFA) catheter is safe, effective, and efficient in achieving pulmonary vein isolation.
- This novel single-shot PFA catheter is capable of ablation as well as performing electroanatomical mapping, resulting in efficient procedures, using single transeptal access.
- Elective protocol-driven remap data revealed a high rate of durability of electrical pulmonary vein isolation.

## Introduction

Catheter ablation is a well-established therapy for the treatment of atrial fibrillation (AF).^1^ The role of thermal energy modalities, for decades the mainstay for catheter ablation, is being challenged by pulsed field ablation (PFA) technologies. Pre-clinical and first-in-human AF ablation studies have demonstrated that this non-thermal ablation approach provides a more efficient and safe ablation procedure.^2–15^ Furthermore, invasive remapping studies have demonstrated that several of these PFA technologies result in high rates (>90%) of durable pulmonary vein isolation (PVI).^9–11,16,17^ Indeed, pivotal trials with several ‘single-shot’ PFA catheters—including the multielectrode pentaspline catheter, the lasso-like curvilinear catheter, and the variable-loop curvilinear catheter—and one focal ablation catheter technology have led to regulatory approval in the USA and/or Europe.^11,18–20^

Despite the optimism surrounding PFA, the post-approval clinical experience has been mixed with regard to PVI durability. In a recent single-centre study of 25 patients undergoing remapping for clinical recurrences, durability was 90.9% on a per-vein basis.^21^ On the other hand, in the multicentre *MANIFEST-PF* and *EUPORIA* registries, in patients undergoing clinically-indicated redo procedures after first-ever AF ablation using the pentaspline PFA catheter, PVI durability was observed in 73% and 71% of pulmonary veins (PVs), translating to only 46% and 38% of patients having all PVs durably isolated, respectively.^22–24^ Some of this reduction in PVI durability is to be expected as (i) the result of transition from first-in-human studies where procedures are typically performed by few operators with the greatest familiarity with the studied device and attention to procedural steps, and (ii) there may remain technical/procedural challenges affecting lesion quality. Indeed, several ‘single-shot’ PFA catheters require serial re-positioning and rotation to generate an overlapping contiguous lesion set to electrically isolate the PVs, and assuring tissue proximity with the ablation electrodes may be challenging depending on the catheter and patient anatomy.

Recently, a novel ‘single-shot’ large-lattice PFA catheter was designed to: (i) be conformable to accommodate varying left atrial and PV anatomies, (ii) avoid serial re-positioning and rotation, thus enhancing its ease of use, (iii) provide all-in-one mapping and ablation via a single transeptal access, and (iv) be linked to a dedicated electroanatomical mapping and magnetic navigational system to support a minimized fluoroscopy approach to the procedure.^25,26^ Herein, we present the first-in-human clinical experience with this large-lattice PFA catheter, including: procedural performance and efficiency, safety, PVI durability upon invasive remapping, and one-year clinical efficacy.

## Methods

### Study design

The objective of this first-in-human, prospective, single-arm, multicentre trial was to evaluate the safety and efficacy of the novel conformable, single-shot PFA catheter (Sphere-360; Medtronic Inc., Minneapolis, MN) in treating patients with paroxysmal AF (PAF). This analysis encompasses the combined datasets of two concurrent trials with largely identical trial designs (NCT05144503 and NCT05115214), with eligible, consenting patients being consecutively enrolled at three centres (two in the Czech Republic and one in Lithuania; see Supplementary material for the list of centres and investigators). Prior to enrolment, each centre received approval from the respective local ethics committees, and informed consent was obtained from all patients. This study was conducted according to the principles outlined in the Declaration of Helsinki. The authors were responsible for data integrity and collection, analyses, and evidence dissemination.

### Patient population

Patients with symptomatic PAF (ages 18–75 years) who were naïve to cardiac catheter ablation and who had failed or were intolerant of at least one Class I–IV antiarrhythmic drug (AAD) were eligible for the study. Within 12 months prior to enrolment, PAF had to be documented by a physician’s note and at least two episodes of AF detected by ECG. Exclusion criteria included body mass index > 40 kg/m^2^, left ventricular ejection fraction (LVEF) < 40%, NYHA Class III or IV heart failure classification, and left atrial diameter > 50 mm. However, patients were not excluded based on PV anatomy or history of atrial flutter (AFL)/atrial tachycardia (AT). Detailed inclusion and exclusion criteria are provided in Supplementary material online, *Table S1*.

Within 30 days before the index ablation procedure, an initial screening was performed to determine eligibility and obtain baseline assessments, including: medical and arrhythmia history, medications, physical exam, transthoracic echocardiogram, 12-lead ECG, and a pregnancy test (as applicable). Thrombus screening was performed within 24 h of the ablation procedure via transoesophageal echocardiography or intracardiac echocardiography (ICE).

### Single-shot pulsed field ablation system

#### System description

**Figure 1:**
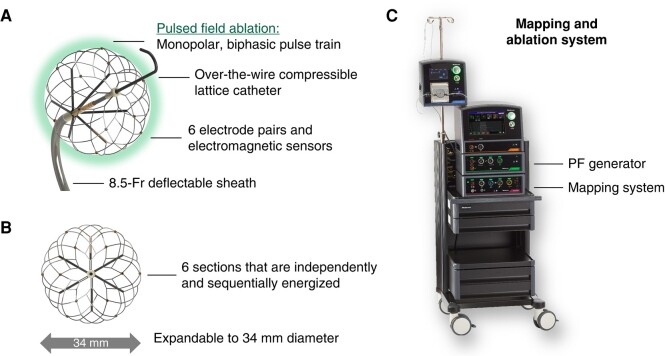
Single-shot pulsed field ablation catheter and pulsed field ablation system. The system includes the (*A* and *B*) single-shot PFA catheter and (*C*) the integrated mapping system, PF and RF generators, and irrigation pump. (*A*) The catheter has an electrode array composed of a compressible nitinol-based lattice that contains six pairs of mini-electrodes. PF energy emanates from the entire lattice framework as the six sections of the lattice are independently and sequentially energized. (*B*) The lattice is expandable up to 34 mm. PF, pulse field; RF, radiofrequency.

The ablation system is composed of three main components, including: (i) the compressible large-lattice circumferential PFA catheter, (ii) the PFA generator, and (iii) the electroanatomical mapping (EAM) system (*Figure 1*). The 8 Fr over-the-wire single-shot PFA catheter (Sphere-360) includes an electrode array comprised of a compressible nitinol lattice framework that is expandable up to 34 mm in diameter.^25,26^ This flexible design allows for the catheter to be utilized to treat diverse PV anatomies. To allow visualization on fluoroscopy, the lattice contains radiopaque makers. Electromagnetic sensors allow the catheter to be rendered in the EAM system (including the HexaMap catheter interface unit and Prism-1 mapping software; Medtronic). Six mini-electrode pairs, which can be used for electrogram recording, impedance measurement, mapping, and cardiac stimulation, are evenly dispersed across the lattice surface.

#### Pulsed field ablation

**Figure 2:**
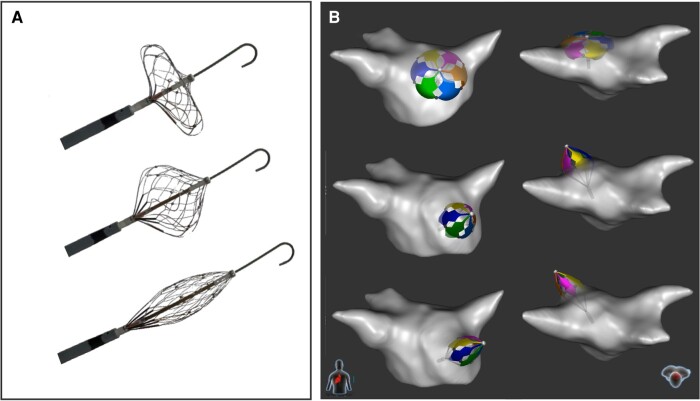
Rendering of left atrial and pulmonary vein anatomies with the PFA catheter. (*A*) The large-lattice catheter is shown along with a J-tip guidewire in various shapes—from a disk/pancake (top), deployed/basket (middle) to elongated (bottom) configurations. (*B*) To render the left atrial and pulmonary vein anatomies, the catheter is simply manoeuvred within the chamber and elongated into each pulmonary vein (see online Supplementary material online, *Video S1* for a video example of this).

For energy delivery, the pulsed field (PF) generator (HexaPulse; Medtronic) delivers a proprietary monopolar biphasic PF waveform between surface dispersive pads and the six (equal area) sections of the electrode array by independently and sequentially energizing these elements that encompass the entire lattice framework of the single-shot PFA catheter (these six sections are represented by the various colours in *Figure 2B*). The waveform is comprised of a train of microsecond pulses ranging from 1.3–2.0 kV that are delivered without cardiac synchronization over 5–6 s. The pulse waveform and system hardware/software were optimized during the trial, resulting in three patient cohorts being evaluated: PULSE1, PULSE2, and PULSE3 (this evolution was not pre-specified, but occurred because of suboptimal durability with initial waveforms). Briefly, for these three pulse waveforms: PULSE1 (5.2 s lesion) had a target of two applications per vein, and PULSE2–3 (5.9 s lesion) had a target of four applications per vein. In order to improve lesion durability, propriety changes were also made to the waveform parameters and vectoring.

#### Electroanatomical mapping

The EAM system employs electromagnetic sensors within the single-shot PFA catheter lattice framework to render its shape. To reduce errors, the EAM systems utilize respiratory gating, and anatomical information is collected simultaneously with the construction of voltage and activation maps. The local impedance of the six mini-electrode pairs can be used to assess the placement of the lattice relative to the cardiac tissue, and bipolar electrograms are measured using the six pairs of mini-electrodes.

### Procedural workflow

**Figure 3:**
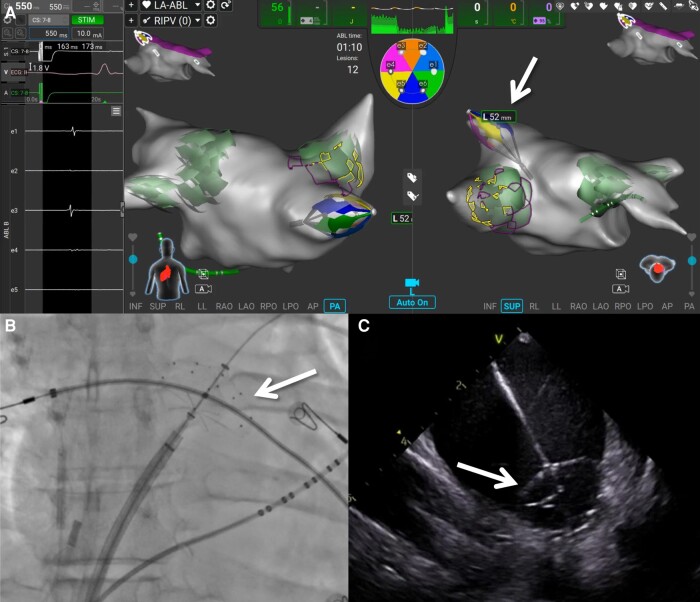
Pulmonary vein isolation using the PFA catheter. (*A*) A screenshot of the electroanatomical mapping system is shown with shadows (green shadows) of where the PFA lesions were placed, and the location of the ablation catheter in the right inferior pulmonary vein (white arrows) in both posterior (left) and superior (right) views. The PFA catheter (white arrows) is also shown at the left superior pulmonary vein antrum in a large basket configuration on fluoroscopy (*B*) and on intracardiac echocardiography (*C*). While not formally analysed, PVs were typically acutely isolated with the first PF application; subsequent successively proximal applications extended the level of isolation and acted as bonus lesions to enhance durability. See online Supplementary material online, *Video S2* for an example of PFA of a PV.

Procedures were typically performed under general anaesthesia, with the administration of paralytic agents per operator discretion. Briefly, intravenous heparin was administered before transseptal puncture, with a target activation clotting time of >300 s. Following transseptal puncture, the single-shot PFA catheter was manoeuvred through an 8.5 Fr steerable sheath (Agilis; Abbott, St. Paul, MN) and advanced over a guidewire into the left atrium. In conjunction with the mapping system, the study device was employed to reconstruct PV and LA anatomies, and PVI was achieved by advancing the extended (collapsed) single-shot PFA catheter into the targeted PV and gradually retracting and expanding the lattice framework under electroanatomical mapping, fluoroscopy, and/or ICE guidance between each application to conform to the shape of the vein (*Figure 2*). Typically, ∼4 applications of biphasic pulses were delivered per PV, with investigators slightly moving the catheter antral between applications (*Figure 3*).

Pulmonary vein entrance block (and exit block, per operator preference) was confirmed after using two alternative testing/challenging methods, either: (i) a 20 min wait period, or (ii) an infusion of adenosine. As an additional assessment of the level of acute isolation, post-ablation EAMs were utilized in some patients using the same catheter. To treat organized AT or AFL (or at an investigator’s discretion), linear lesions were permitted using a focal 9 mm lattice-tip catheter (Sphere-9; Medtronic), and either RF or PF energy as appropriate according to the IFU recommendations.^11,27^

### Follow-up

All patients were monitored throughout the 12-month follow-up period, including in-person or virtual visits at 10 days, 75 days, 6 months, and 12 months post-ablation. Following the 90-day blanking period, transtelephonic monitoring (TTM) was conducted weekly through 21 weeks, and monthly thereafter. Patients also recorded TTMs throughout the study whenever symptomatic, and continuous 48 hour Holter monitoring was performed at 6 and 12 months. Of note, a few of the latter patients also received implanted cardiac monitors (LinQ; Medtronic), but these patients did not contribute to the long-term follow-up outcomes (just exited the blanking period).

At investigator discretion, optional oesophagogastroduodenoscopy (EGD) and brain magnetic resonance imaging (MRI) were conducted 24–48 h after the procedure. MRI scans were reviewed by an independent neuroradiologist to identify acute ischaemia based on diffusion-weighted imaging (DWI) hyperintensity with corresponding apparent diffusion coefficient (ADC) reduction, with or without T2 fluid-attenuated inversion recovery (FLAIR) hyperintensity.^28–31^

At 75 ± 15 days post-ablation, an elective invasive remapping procedure was scheduled. If patients were remapped, an additional virtual visit was conducted 10 ± 3 days following the remap procedure. Any electrical reconnection in the PVs or linear lesions was permitted to be re-ablated with a commercially available radiofrequency ablation catheter during the remapping procedure.

### Study efficacy and safety outcomes

The primary efficacy outcome was acute electrical isolation of all the PVs utilizing the single-shot PFA catheter. Secondary efficacy outcomes included chronic efficacy, defined as freedom from >30 s atrial arrhythmia (AF/AFL/AT) recurrence beyond the 90-day blanking period, and PVI durability on a per-vein (veins that remain isolated) and per-patient (patients with all PVs isolated) basis.

The primary safety outcome was the incidence of study device-related serious adverse events (SAEs) within 7 days, including: death, myocardial infarction, persistent phrenic nerve palsy, transient ischaemic attack (TIA), stroke, thromboembolism, major vascular complications/bleeding, heart block, gastroparesis, severe pericarditis, hospitalization (initial and prolonged) due to cardiovascular or pulmonary adverse events (AEs), cardiac tamponade/perforation (up to 30 days), PV stenosis (up to 180 days), and atrio-esophageal fistula (up to 180 days). The secondary safety outcome was the proportion of subjects experiencing device- or procedure-related SAEs as assessed at each follow-up visit. All AEs and complications were adjudicated by an independent data safety monitoring board/clinical endpoint committee (see Supplementary material).

### Statistical analysis

Patients received either PULSE1, PULSE2, or PULSE3 sequentially as they enrolled in the study. To improve lesion durability, the parameters of the pulse waveforms and hardware/software updates were made over the study enrolment, ending with the most optimized PULSE3 cohort. The study cohorts included the total cohort (PULSE1, PULSE2, and PULSE3 pooled data) and the PULSE3 cohort. Being a first-in-human study, there was no formal hypothesis testing or power calculation. *Post hoc* analyses compared PULSE3 vs. the combined PULSE1/PULSE2 data to elucidate the differences observed with waveform evolution and optimization of the PFA system.

Continuous variables are summarized as mean ± SD and comparisons between PULSE3 and PULSE1/PULSE2 were made with two-sample *t*-tests. Categorical variables are summarized as number and percentage, and these data were compared between groups with a Fisher’s exact test. Freedom from secondary efficacy failure (recurrence of AT/AF/AFL post 90-day blanking period) was estimated using Kaplan–Meier methods. A log-rank test was utilized to compare secondary efficacy between PULSE3 and PULSE1/PULSE2.

## Results

### Study population

**Figure 4:**
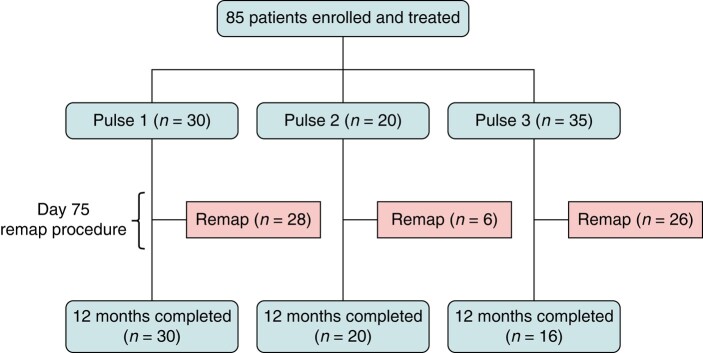
Patient flow diagram. The overall patient population and individual PULSE1 (*n* = 30), PULSE2 (*n* = 20), and PULSE3 (*n* = 35) cohorts are shown, along with the number of patients in each treatment cohort that underwent invasive remapping and reached 12-month follow-up.

At three centres, including five operators, a total of 85 PAF patients underwent PFA using the PULSE1 (*n* = 30), PULSE2 (*n* = 20), or PULSE3 (*n* = 35) waveform (*Figure 4*). At the time of this analysis, all PULSE1 and PULSE2 patients had reached the 1-year follow-up point, while in the PULSE3 cohort, 16 patients reached one-year follow-up, 15 patients had reached 75-day follow-up.

Analysis cohorts were defined as all patients (total cohort; *n* = 85) and patients in the PULSE3 cohort (PULSE3; *n* = 35). Baseline characteristics for the total cohort and PULSE3 are shown in *Table 1*. The overall patient population was 56% male, 58.3 ± 9.8 years old, and diagnosed with PAF for 3.4 ± 3.8 years. The total and PULSE3 cohorts were similar in baseline characteristics, including CHA_2_DS_2_-VASc scores (1.8 ± 1.3 vs. 1.8 ± 1.2), hypertension history (71% vs. 69%), left atrial diameter (41.2 ± 4.2 vs. 41.5 ± 3.9 mm), and LVEF (60.3 ± 5.9% vs. 61.7 ± 6.3%). Over 90% of patients in both groups were receiving oral anticoagulant medications before the procedure. All patients had received some Class I–IV AADs pre-ablation, with 78% of the total cohort and 74% of the PULSE3 group taking Class I/III AADs at baseline.

**Table 1:** Baseline patient characteristics

Characteristic	Total cohort (*n* = 85)	PULSE3 (*n* = 35)	*P*-value^a^
Age (years)	58.3 ± 9.8	58.1 ± 11.3	0.85
Male sex	48 (56)	25 (71)	0.03
Time from AF diagnosis to enrolment (years)^b^	3.4 ± 3.8	2.6 ± 3.6	0.10
Body mass index (kg/m^2^)	29.4 ± 4.3	29.1 ± 4.2	0.68
LA diameter (mm)	41.2 ± 4.2	41.5 ± 3.9	0.52
LVEF (%)^c^	60.3 ± 5.9	61.7 ± 6.3	0.07
CHA_2_DS_2_-VASc^d^	1.8 ± 1.3	1.8 ± 1.2	1.00
Prior DC cardioversion for AF	40 (47)	15 (43)	0.66
Hypertension	60 (71)	24 (69)	0.81
Diabetes (type 2)	6 (7)	4 (11)	0.22
Stroke or TIA	3 (4)	1 (3)	1.00
Coronary artery disease	8 (9)	4 (11)	0.71
Sleep apnoea	2 (2)	0 (0)	0.51
Atrial tachycardia	7 (8)	4 (11)	0.44
Atrial flutter	15 (18)	6 (17)	1.00
Prior CTI ablation^e^	1 (7)	0 (0)	1.00
Medications			
Anticoagulant use for ≥3 weeks pre-ablation	79 (93)	34 (97)	0.39
Warfarin	2 (2)	0 (0)	0.51
DOAC	76 (89)	33 (94)	0.30
Other	1 (1)	1 (3)	0.41
Prior AADs^f^			
Classes I–IV	84 (100)	34 (100)	NA
Class I or III	66 (78)	26 (74)	0.79

Values are reported as mean ± SD, incidence number (percentage).

AF, atrial fibrillation; AADs, antiarrhythmic drugs; DC, direct current; DOAC, direct oral anticoagulant; LA, left atrium; LVEF, left ventricular ejection fraction.

^a^These *P*-values are comparison between the aggregate PULSE1/PULSE2 vs. PULSE3 cohorts.

^b^80 patients with time from AF diagnosis to enrolment reported; 80 total cohort, 35 PULSE3.

^c^84 patients with LVEF reported; 84 total cohort, 34 PULSE3.

^d^84 patients with CHA_2_DS_2_-VAS_c_ reported; 84 total cohort, 34 PULSE3.

^e^Percentage out of patients with atrial flutter.

^f^84 patients with prior AADs reported; 84 total cohort, 34 PULSE3.

### Procedural characteristics

Procedural characteristics are presented in *Table 2*. The procedure time (inclusive of the protocol-mandated adenosine challenge that was employed in 74.2% of PVs) and fluoroscopy time were 56.5 ± 21.6 and 5.7 ± 3.9 min, respectively. The left atrial dwell time for the PFA catheter was 19.1 ± 9.3 min, including both mapping and ablation.

**Table 2:** Procedural characteristics

Characteristic	Total cohort (*n* = 85)	PULSE3 (*n* = 35)	*P*-value^a^
Total procedure time (min)^b^	56.5 ± 21.6	59.3 ± 20.0	*P* = NS
Total fluoroscopy time (min)^c^	5.7 ± 3.9	6.1 ± 3.1	*P* = NS
Single-shot PFA left atrial dwell time (min)^d^	19.1 ± 9.3	22.3 ± 9.0	*P* < 0.05
PVI transpired time (min)^e^	10.0 ± 6.0	10.6 ± 6.3	*P* = NS
Single-shot PFA PVI application time (min)^f^	1.4 ± 0.5	1.8 ± 0.5	*P* < 0.05
General anaesthesia use	84 (99)	34 (97)	*P* = NS
Acute PVI success	335 (100)	134 (100)	*P* = NS
Number of applications per PV	3.9 ± 1.4	4.8 ± 1.3	*P* < 0.05
Non-PVI ablation	16 (19)	6 (17)	*P* = NS
CTI	10 (12)	5 (14)	
Acute non-PVI success	20 (100)	6 (100)	*P* = NS
Pre-ablation map^g^	85 (100)	35 (100)	*P* = NS
Performed with single-shot PFA catheter	80 (94)	34 (97)	
Performed with focal lattice-tip catheter	9 (11)	4 (11)	
Post-ablation map^g^	55 (65)	30 (86)	*P* < 0.05
Performed with single-shot PFA catheter	31 (36)	25 (71)	
Performed with focal lattice-tip catheter	27 (32)	8 (23)	

CTI, cavotricuspid isthmus; LA, left atrium; LCPV, left common pulmonary vein; LIPV, left inferior pulmonary vein; NS, not significant; PFA, pulsed field ablation; PV, pulmonary vein; PVI, pulmonary vein isolation; RIPV, right inferior pulmonary vein; RMPV, right middle pulmonary vein; RSPV, right superior pulmonary vein.

Values are reported as mean ± SD, incidence number (percentage).

^a^ *P* < 0.05 when compared to PULSE1/PULSE2 arm (*n* = 50).

^b^Total procedure time is defined as first sheath inserted to last sheath pulled out and includes the protocol-mandated 20 min wait period or infusion of adenosine.

^c^Total fluoroscopy time is defined as fluoroscopy time through total procedure, vein access to sheath removal.

^d^Single-shot PFA left atrial dwell time is defined as first single-shot PFA catheter inserted to last single-shot PFA catheter pulled out and includes mapping time. Of note, more post-ablation maps were performed in the PULSE3 cohort.

^e^PVI transpired time is defined as first PV ablation to last PV ablation (last PV ablation excludes any PV touch-up after any linear lesions).

^f^Single-shot PFA PVI application time is defined as the time the generator is delivering PF energy during PVI with the single-shot PFA catheter.

^g^Some patients were mapped with both the single-shot PFA catheter and the focal lattice-tip catheter.

The transpired ablation time for PVI, defined as the time elapsing between the beginning of the first lesion and the end of the last lesion, was ∼10 min in both groups (*Table 2*). Overall, 1.4 ± 0.5 min of PF energy was delivered to accomplish PVI in the total cohort, with less delivery time being required for the PULSE1/PULSE2 group (1.2 ± 0.4 s) in comparison to PULSE3 (1.8 ± 0.5 min, *P* < 0.01). An average of 3.9 ± 1.4 applications was delivered per PV in the study population. As the system evolved, applications per PV increased from 3.2 ± 1.0 to 4.8 ± 1.3 for PULSE1/PULSE2 and PULSE3 patients, respectively (*P* < 0.01). Employing only the large-lattice catheter, acute PVI was successful in 100% of the 335 targeted PVs, including 6 right middle PVs and 11 left common PVs; specifically, entrance block was confirmed in all PVs, and exit block was assessed (and confirmed) in 251 of 326 PVs (77.0%). A few selected examples of the varying PV anatomies are shown in Supplementary material online, *Figure S1*.

### Safety

#### Serious adverse events

There were no primary safety events. Specifically, as shown in *Table 3*, there were no occurrences of death, phrenic nerve palsy, cardiac tamponade/perforation, or atrio-esophageal fistula. Only one serious procedure/device-related adverse event occurred: a patient experienced diplopia and vertigo on the day of discharge. There was full symptom remission within 2 days since onset, and both an immediate brain CT and an MRI obtained 9 days from symptom onset ruled out an embolic event. A list of all adverse events is shown in the Supplementary material online, *Table S2*.

**Table 3:** Adverse event incidence

	Adverse event	Number of adverse events (*n* = 85)
SAEs included in the primary safety endpoint	Total subjects with primary safety event	0
	Death	0
	Myocardial infarction	0
	Persistent phrenic nerve palsy	0
	Transient ischaemic attack (TIA)	0
	Stroke/cerebrovascular accident (CVA)	0
	Thromboembolism	0
	Major vascular access complications/bleeding	0
	Heart block	0
	Gastroparesis	0
	Severe pericarditis	0
	Hospitalization (initial and prolonged) due to cardiovascular of pulmonary AE^a^	0
	Cardiac tamponade/perforation^b^	0
	Pulmonary vein stenosis^c^	0
	Atrio-esophageal fistula^c^	0
Serious procedure-related adverse events	Diplopia and vertigo^d^	1 (1.2)

Values are reported as incidence number (percentage).

AE, adverse events; SAEs, serious adverse events.

^a^Excludes hospitalization due to AF/AFL/AT recurrence.

^b^Occurring up to 30 days after index ablation is considered a primary safety event.

^c^Occurring up to 180 days after index ablation is considered primary safety event.

^d^PULSE2 subject. Symptom remission in 2 days. Brain CT and MRI evaluation ruled out an embolic event.

#### Oesophagogastroduodenoscopy

Post-procedure EGD was performed in 18 (21%) patients at 24–48 h following the index ablation; these were performed routinely and not for symptoms. Three oesophageal injuries were observed (minor erythema, mucosal injury, and post-anaesthesia intubation irritation), but all were adjudicated as non-thermal and not device-related, and all resolved without sequelae. Only the intubation finding was procedure-related (*Table 4*). No thermal injuries were noticed.

**Table 4:** Summary of prospective safety assessments

Safety assessments	Total cohort (*n* = 85)
Oesophageal observations	
Patients with assessment	18 (21)
Minor erythema^a^	1 (6)
Injury to mucosa^a^	1 (6)
Post-anaesthesia intubation irritation^b^	1 (6)
Thermal injuries	0 (0)
Brain MRI findings	
Patients with assessment	40 (47)
Acute ischaemia with FLAIR hyperintensity^c^	4 (10)
Acute ischaemia without FLAIR hyperintensity^c^	3 (8)

Values are reported as incidence number (percentage).

ADC, apparent diffusion coefficient; DWI, diffusion-weighted imaging; FLAIR, fluid-attenuated inversion recovery; SCL, silent cerebral lesion.

^a^Not device- or procedure-related.

^b^Procedure-related.

^c^Mutually exclusive.

#### Brain MRI screening

At 24–48 h post-procedure, 40 (47%) patients, with 19 patients from the PULSE3 cohort, underwent brain MRI screening. These MRIs were performed routinely, and not for symptoms. Acute ischaemia (defined as having DWI and ADC congruence) with corresponding T2 FLAIR hyperintensity was identified in four (10%) patients. MRIs with DWI-positivity, but without corresponding T2 FLAIR hyperintensity, were identified in three (8%) patients (*Table 4*). All aforementioned MRI detected brain lesions were asymptomatic and required no medical intervention.

### Antiarrhythmic medications post-ablation

Antiarrhythmic drug therapy was administered to patients at investigator discretion throughout the study. Overall Class I/III AAD usage decreased between Days 90 and 365 in the overall (30–14%) and PULSE3 cohorts (38–13%, *Table 5*). For patients treated with PULSE3, there was no new initiation of Class I/III AADs between Day 90 and Day 365.

**Table 5:** Antiarrhythmic medications during follow-up

Time point	Total cohort (*n* = 66^a^)	PULSE3 (*n* = 16)
Baseline		
None	0 (0)	0 (0)
Class I or III	54 (82)	14 (88)
Day 90		
None	18 (27)	4 (25)
Class I or III	20 (30)	6 (38)
Day 365		
None	27 (41)	5 (31)
Class I or III	9 (14)	2 (13)

Values are reported as incidence number (percentage). No statistical differences observed in AAD utilization between PULSE3 and PULSE1/PULSE2 cohorts.

^a^Paired analysis. Only subjects with 12-month follow-up included (66 of 85 subjects through 12 months).

### Lesion durability

**Figure 5:**
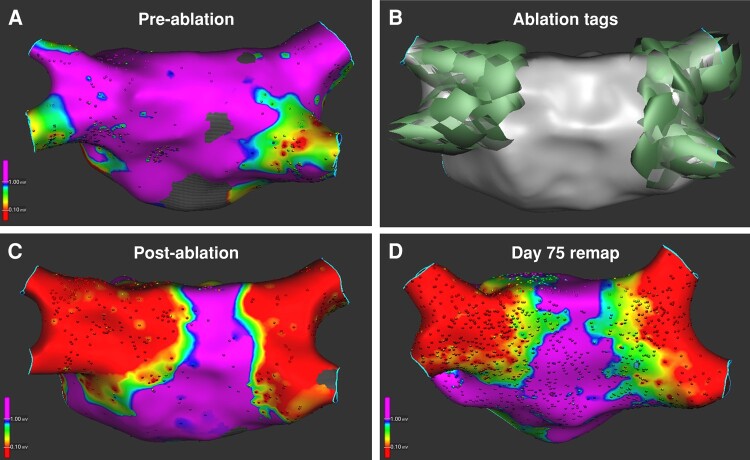
Electroanatomical maps of different phases of pulsed field ablation treatment. Shown are bipolar voltage amplitude maps from three different patients (*A*) before and (*C*) after PFA treatment and from (*D*) Day 75 remap procedure. In (*B*), shadows of the PFA catheter are captured to denote the locations of each of the four PFA applications at each PV. The colour range is 0.1 (red)–1.0 mV (purple).

At 87.8 ± 71.1 days following the index ablation, 60 patients (71%), including 26 (74%) from the PULSE3 cohort, presented for the optional invasive remapping procedure to assess ablation durability (*Figure 4*). Typical voltage maps representing PV electrical activity pre-ablation, immediately post-ablation and at 75-day remap are illustrated in *Figure 5* (note the level of electrical isolation was antral, and not just ostial).

**Figure 6:**
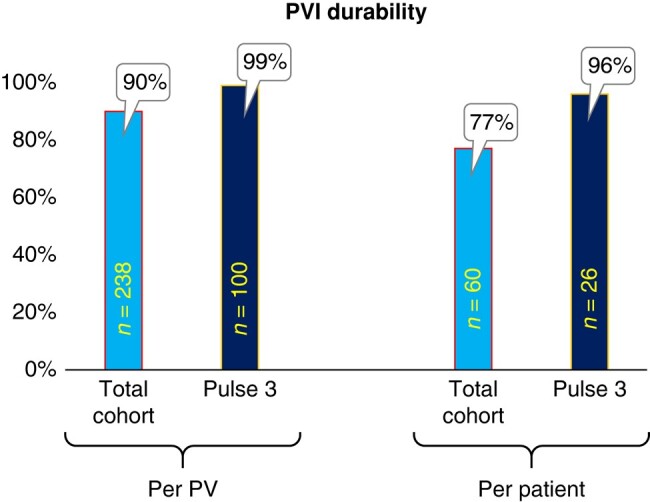
Durability of pulmonary vein isolation on invasive remapping. PVI durability for patients presenting for remapping from the total cohort (*n* = 60; bright blue bars) or the PULSE3 cohort (*n* = 26; dark blue bars), with 238 and 100 remapped veins, respectively. PVI durability is shown on a per-vein (left) or per-patient basis (right). The *P*-value comparing PULSE1/PULSE2 (*n* = 34) vs. PULSE3 (*n* = 26) is <0.01 for both per-vein and per-patient durability. PV, pulmonary vein; PVI, pulmonary vein isolation.

During the remap procedures, 24 of the 238 PVs (10%) were found to be electrically reconnected. The vast majority of these PV reconnections (23 of 24) occurred in the PULSE1/PULSE2 cohort, with only one PV being reconnected in a PULSE3 patient (*P* < 0.01; *Figure 6*). On a per-vein basis, the overall PVI durability rate was 90%, translating to 77% of patients having all veins durably isolated. However, waveform optimization resulted in a significantly improved per-vein durability of 99% and per-patient durability of 96% with PULSE3 treatment, respectively (*P* < 0.01 for both: *Figure 6* and Supplementary material online, *Figure S2*). Outcomes by centre are shown in Supplementary material online, *Table S3*.

During the remapping procedure, one patient in PULSE3 and 16 patients in the total cohort were retreated (details in Supplementary material online, *Table S4*). Of these 16 patients, 15 received re-ablation of their PVs, with two patients receiving linear lesion touch-up. For the one PULSE3 patient with a PV reconnection, there was a single delayed electrogram at the anterior aspect of the left superior PV (see Supplementary material online, *Figures S3 and S4*). This PV was treated with RF energy delivery to this location.

### One-year efficacy

**Figure 7:**
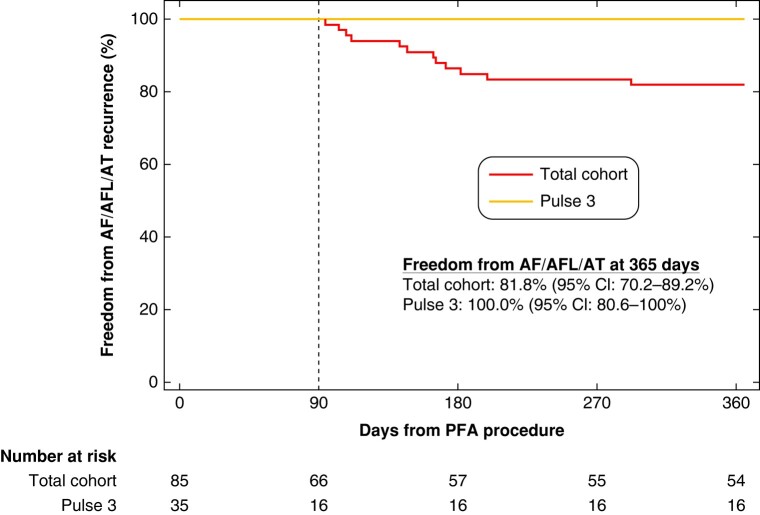
Freedom from atrial arrhythmia recurrence at 365 days. Shown are the Kaplan–Meier estimates of freedom from ≥30 s recurrence of AF/AFL/AT at 365 days in the total cohort (red line) and PULSE3 cohort (gold line). Note that due to no events observed in the PULSE3 cohort, the 95% confidence interval is calculated with Wilson’s method for binomial proportions. Comparing PULSE1/PULSE2 (*n* = 50) vs. PULSE3 (*n* = 16), *P* = 0.04 (log-rank test). The dotted line denotes the end of the 90-day blanking period. AF, atrial fibrillation; AFL, atrial flutter; AT, atrial tachycardia.

The mean follow-up for the full cohort was 290 ± 145 days, with 81% of the full cohort reaching the 12-month endpoint (*Figure 7*). There was good adherence to the monitoring protocol with compliance rates of 96.3% and 100% for TTM and Holter monitoring, respectively. After the 90-day blanking period, the 1-year freedom from AF/AFL/AT recurrence was 81.8% (95% CI 70.2–89.2%) for the total cohort, 76.0% (95% CI 61.6–85.6%) for the PULSE1/PULSE2 aggregate cohort, and 100% (95% CI 80.6–100%) for the PULSE3 cohort (*Figure 7*).

## Discussion

In a first-in-human, single-arm trial of catheter ablation for patients with PAF, we assessed the safety and efficacy of PVI using a single-shot PFA catheter and a dedicated electroanatomical mapping system. The primary findings are: (i) using an average of 3.9 PFA applications per PV, acute PVI was achieved in 100% of the cohort; (ii) the procedure was efficient with a mean left atrial dwell time of 19 min, an average transpired PVI ablation time of 10 min, and a mean fluoroscopy time of 6 min; (iii) no observed primary safety events; (iv) upon invasive remapping, lesion durability improved such that the optimized waveform (PULSE3) resulted in 99% per-vein PFA lesion durability; and (v) high one-year freedom from atrial arrhythmia recurrence—82% and 100% for the total and PULSE3 cohort, respectively. Together, these findings suggest that the conformable single-shot PFA catheter in conjunction with a novel electroanatomical mapping system is a highly efficient, safe, and effective tool for PVI in patients with PAF.

### Pulsed field lesion optimization with tissue proximity

For thermal ablation, there are a wealth of biophysical studies over the past 2–3 decades that have elucidated strategies to optimize the delivery of either RF energy (e.g. saline irrigation, contact force sensing, and surface temperature monitoring) or cryothermal energy (e.g. uniform distribution of the refrigerant and minimization of ambient blood flow) In contrast, our understanding of the parameters that optimize PFA is still nascent—perhaps in part because the PF waveforms utilized by various technologies are proprietary and thus unknown to most practitioners and investigators.

However, *it is known* that for each PFA catheter, the electrode array generates an electrical field of a certain magnitude and geometry, and to have an ablative effect, this electrical field must project into the target tissue. Accordingly, tissue proximity of the ablation PF electrodes with the target tissue is necessary. And it is possible that electrode-tissue contact may further enhance tissue penetration of the electrical field, since boundaries of various conductivity may alter the penetration of an electrical field. Indeed, in an *ex vivo* PFA study in which a linear prototype catheter was either in contact with or suspended above the ventricular epicardial surface in an isolated porcine heart model, the relationship between lesion depth and offset distance was linear: the deepest lesions were created with 0 mm offset distance, and any offset distance simply reduced the lesion depth by that *same offset*.^32^ On the other hand, the importance of contact force is less clear—with some studies identifying no significant relationship between contact force and lesion dimensions, and others suggesting that such a relationship does exist.^33,34^

The large-lattice design allows for compliance of the catheter framework, so there is considerable apposition of the ablative elements with the target left atrial and PV tissue when positioned at the PV ostia. Given this tissue proximity, it is unsurprising that the large-lattice PFA catheter would result in not just an efficient acute PVI procedure, but also such durable ablation lesions. Not only is proximity optimized by the conformability of the lattice, but the extent of interaction of the lattice with the tissue allows for longitudinal redundancy of the PV isolating lesion. As with many PFA technologies, even with this large-lattice catheter, late durability improved considerably with successive improvement to the PF waveform, though it is notable that even with the initial PULSE1 waveform, 82% of the PVs were found to be durably isolated. But ultimately, the PULSE3 waveform achieved 99% PV durability—translating to 96% of patients with all PVs durably isolated. This is of particular notice since there were no anatomical screening strategies employed to eliminate ‘unfavourable’ anatomical LA–PV variants.

These procedures were performed by five different operators, raising the possibility that the high rate of durability observed in this study might be transferable to general clinical practice. Indeed, as noted above, variable lesion durability has been observed in clinical practice with the pentaspline catheter, so it is unknown how this lattice catheter will perform.^21–24^ Accordingly, future clinical studies with more operators from a variety of clinical centres are necessary.

### The value of the electroanatomical mapping system

Since the large-lattice catheter is equipped with electromagnetic sensors, it allows rapid 3D reconstruction of the LA and PV anatomies (*Figure 2*, Online Supplementary material online, *Video S1*). Six mini-electrode pairs collect local electrograms and provide electrogram information. Such an integration with a 3D electroanatomical mapping system enables intuitive positioning of the lattice catheter with minimal need for fluoroscopy. Recording of PF applications as green shadows helps to optimize deployment of lesions within the PVs and at the PV antra. Fast remapping after ablation allows an estimation of the level of PVI. By combining all of these capabilities into a single catheter, there are important potential advantages to workflow, procedural cost, and perhaps even safety (since catheter exchanges would be minimized). However, in this study, a no-exchange approach was employed in only a subset of patients; future studies dedicated to this approach are necessary to fully assess this strategy.

### Clinical effectiveness of the conformable pulsed field ablation catheter

The high degree of lesion durability translated to favourable clinical effectiveness, with a 1-year clinical success rate of 81.8% in the full cohort. It may be that this high degree of success is a reflection of a proximal/antral level of electrical isolation—a morphological observation that was both observed in the acute post-PFA electroanatomical voltage map, and maintained in the subsequent remapping procedure (*Figure 5*). It may also be important to better understand and characterize the impact of PFA on the peri-atrial autonomic ganglia with this large-lattice catheter. We and others have previously shown that PFA using various catheter technologies may have differential effects on atrial myocardium and adjacent ganglia: often with sparing of the ganglia.^35–38^ However, less effect on the ganglia does not equal zero effect—and it may be that these adjunctive effects may modulate clinical success beyond that predicted simply by the durability of PVI.^39^

It is intriguing that those patients undergoing ablation with the PULSE3 waveform demonstrated 100% clinical success. But any enthusiasm must be tempered by the relatively small number of patients that completed follow-up. Larger prospective multicentre studies of this waveform with this large-lattice PFA catheter should be conducted—perhaps with greater attention focused on an AF burden endpoint.

### Safety of the conformable pulsed field ablation catheter

There were no major safety events observed in this clinical study. Most importantly, the tissue preferentiality characteristic of PF energy was reflected by the absence of atrio-esophageal fistula or other oesophageal complication. In the subset of patients undergoing routine post-procedure EGD, there was no evidence of oesophageal ablative effects. And as is typical for PFA studies, it should be noted that no oesophageal protection or monitoring strategies were employed. There was also no evidence of phrenic nerve injury or PV stenosis. Regarding the latter, while routine follow-up cardiac CT or MRI were not performed, 71% of the 85-patient cohort did undergo repeat electroanatomical mapping, and there were no observed instances of PV stenosis.

In the post-approval experience of the most commonly employed PFA catheter, the pentaspline PFA catheter, the major complication seen was pericardial tamponade—but no such tamponade occurred with this large-lattice catheter.^15,22,24,40^ This likely results from the confluence of several important attributes of this technology: (i) an over-the-wire design to facilitate atraumatic PV ostial entry, (ii) the compliant lattice framework, (iii) the absence of complex catheter manipulation/rotation/etc., and (iv) guidance by an integrated electroanatomical mapping system.

There were also no strokes or TIAs observed in this cohort. The fact that the catheter is advanced through a conventional 8.5 Fr sheath, and the all-in-one mapping and ablation design minimizing the need for catheter exchange, likely minimizes the possibility of inadvertent air introduction into the circulation—an issue reported in early experience with other larger catheter technologies.^15,22,24^ And upon routine brain MRI, the rate of silent cerebral ischaemic events and lesions was well within the range reported for other PF and thermal ablation technologies.^15–19,28–31^ However, we did not perform high-resolution DWI, an approach shown in the AXAFA-AFNET5 trial to increase lesion detection.^30^

Recent data has indicated that PFA-related haemolysis could result in acute kidney injury as a direct function of the number of PF applications.^40,41^ This was not observed in our study, and no mitigation strategies were employed since we were not aware of this potential complication when most of the procedures in this study were performed.

### Limitations

Patients were consecutively enrolled in this study. Thus, the consecutive refinements of the pulse waveform led to the PULSE3 cohort being the most incomplete in full reporting of one-year outcomes, so the reported 100% success should be viewed provisionally. On the other hand, 74% of the PULSE3 patients underwent the remapping procedure, so the durability data is quite robust. Secondly, given the non-randomized nature of this first-in-human study, one must be careful about comparing the one-year outcomes with other ablation technologies: outcomes are highly susceptible to variability in the patient cohorts (e.g. baseline AF burden and comorbidities) and intensity of follow-up ECG monitoring. Thirdly, these procedures were performed by experienced operators, so larger multicentre studies are needed to assess generalizability. For example, in the *FARA-Freedom* post-market multicentre registry of the pentaspline catheter, the primary success rate of 66.6% was less than that observed in the initial first-in-human studies of this technology.^8,9,42^ Fourthly, several of the safety assessments (brain MRI, EGD) were optional in this first-in-human study, so a bias may have been introduced by this heterogeneity; future studies dedicated to these endpoints are needed. Fifthly, ICE was employed in all cases so outcomes without ICE need to be assessed. Sixthly, future pre-clinical and clinical studies are needed to fully characterize any thermal effect of the PULSE3 waveform. Finally, only pAF patients were included in this clinical trial. With regard to a persistent AF population, it remains to be demonstrated whether this PFA catheter could create lesion sets beyond PVI, and what would be the resulting efficacy and safety profile.

## Conclusion

In this first-in-human experience, PVI utilizing a conformable single-shot PFA catheter and a dedicated electroanatomical mapping system was efficient, safe, and effective for the treatment of patients with PAF, with durable lesions confirmed by invasive remapping.

## Supplementary Material

euae090_Supplementary_Data

## Data Availability

The primary data used in the research and analysis are not available.
