# Supplementary material for: First-in-human clinical series of a novel conformable large-lattice pulsed field ablation catheter for pulmonary vein isolation
Source: Europace. 2024 Apr 8;26(4):euae090. doi: 10.1093/europace/euae090 (PMC11057205; doi:10.1093/europace/euae090)
Supplement: euae090_Supplementary_Data [file euae090_supplementary_data.zip › Sphere-360 FIH Manuscript_Suplement_v2.0 - Clean.docx]

**SUPPLEMENTAL MATERIAL**

**First-in-Human Clinical Series of a Novel Conformable Large-Lattice Pulsed Field Ablation Catheter for Pulmonary Vein Isolation**

Reddy VY, Anter E, Peichl P, et al.

Table of Contents:

Page 2: List of Sites/Investigators

Page 3: Clinical Events Committee Membership

Page 4-5: Table S1. Inclusion / Exclusion Criteria

Page 6: Table S2: All Adverse Events

Page 7: Table S3: Outcomes by Center

Page 8: Table S4: Reconnected Pulmonary Veins During the Remap Procedure.

Page 9: Figure S1: Diverse Pulmonary Vein Anatomies Treated with the Single-shot Pulsed Field Ablation Catheter

Page 10: Figure S2: Durability of PV Isolation of all Three Pulsed Field Waveforms

Page 11: Figure S3: PULSE3 Patient with a PV Reconnection: Electroanatomical Mapping

Page 12: Figure S4: PULSE3 Patient with a PV Reconnection: Mapping & Ablation

**List of Sites/Investigators**

Participating sites, principal investigators, and sub-investigators are included in the table below.

| Site Name and Address | Principal Investigator | Sub-Investigators |
| --- | --- | --- |
| Nemocnice Na Homolce Hospital (Homolka) Roentgenova 37/2 150 30 Praha 5 Czech Republic | Peter Neuzil, MD, PhD, FESC | Jan Škoda, MD  Pavel Hala, MD  Tereza Stastna, MD  Veronika Svecova, MD  Jan Petru, MD  Libor Dujka, MD  Milan Chovanec, MD  Vivek Reddy, MD  Branislav Chudiak, MD |
| Institut klinické a experimentální medicíny (IKEM) Vídeňská 1958/9 140 21 Praha 4 Czech Republic | Josef Kautzner, MD, PhD | Petr Peichl, MD, PhD  Pedrag Stojadinovic, MD |
| Vilniaus universiteto ligoniné  Santaros Klinikos (Vilnius)  University Hospital  Santariškių str. 2  08661 Vilnius  Lithuania | Gediminas Rackauskas, MD, PhD | Diana Sudaviciene, MD  Sigita Aidietiene, MD, PhD Jurate Barysiene, MD, PhD  Ieva Stundiene, MD  Justinas Bacevicius, MD  Arturas Samuilis, MD  Audrius Aidietis MD, PhD  Germanas Marinskis, MD, PhD Mindaugas Mataciunas, MD |

**Clinical Events Committee Membership**

The Clinical Events Committee (CEC) /Data and Safety Monitoring Board (DSMB) is listed below.

| **Institution and Address** | **CEC/DSMB Member** |
| --- | --- |
| Brigham and Women’s Hospital  2301 Erwin Road  Durhan, NC 22710  United Sates of America | Bruce A. Koplan, MD - Chair |
| University of Utah School of Medicine  211 Mountain Avenue  Springfield, NJ 07081  United States of America | T. Jared Bunch, MD |
| Baim Institute for Clinical Research  85 Stockdale Road  Needham, MA 02492  United States of America | Matthew Reynolds, MD, MSc |

**Table S1. Inclusion / Exclusion Criteria**

| **Inclusion Criteria**  Subjects must satisfy all of the following inclusion criteria to be included in the study: |
| --- |
| 1. History of symptomatic paroxysmal atrial fibrillation (PAF) documented by:  - A physician’s note indicating recurrent, self-terminating AF. - At least two electrocardiographically documented AF episodes within 12 months prior to enrollment  1. Failure or intolerance of at least one Class I-IV antiarrhythmic drug 2. Suitable candidate for catheter ablation. 3. Age ≥ 18 and < 75 years. 4. Subject is able and willing to give informed consent. 5. Subject is able and willing, and has ample means to comply with all pre-procedure, post-procedure and follow-up testing requirements. |
| **Exclusion Criteria**  Subjects are not eligible if any of the following exclusion criteria are present: |
| 1. Continuous AF lasting more than 7 days. 2. AF secondary to electrolyte imbalance, thyroid disease, acute alcohol intoxication, or reversible or non-cardiac cause. 3. Previous left atrial catheter or surgical ablation (including septal closure or left atrial appendage closure). 4. Valvular cardiac surgical/percutaneous procedure (e.g., ventriculotomy, atriotomy, and valve repair or replacement and presence of a prosthetic valve). 5. Any carotid stenting or endarterectomy. 6. Any cardiac procedure (surgical or percutaneous) or percutaneous coronary intervention within 90 days prior to the initial procedure. 7. Coronary artery bypass graft (CABG) procedure within the 6 months prior to the index ablation procedure. 8. Awaiting cardiac transplantation or other cardiac surgery within 12 months following the initial ablation procedure. 9. Presence of any type of implantable cardiac defibrillator (with or without biventricular pacing function). 10. Documented thromboembolic event (stroke or transient ischemic attack) within the past 6 months (180 days). 11. Documented left atrial thrombus on imaging. 12. History of blood clotting or bleeding abnormalities. 13. Any condition contraindicating chronic anticoagulation. 14. Myocardial infarction (MI) within the 3 months (90 days) prior to the initial procedure. 15. Body mass index >40 kg/m2. 16. Left atrial diameter >50 mm (anteroposterior). 17. Diagnosed atrial myxoma. 18. Left ventricular ejection fraction (EF) < 40%. 19. Uncontrolled heart failure or NYHA Class III or IV heart failure. 20. Rheumatic heart disease. 21. Hypertrophic cardiomyopathy. 22. Unstable angina. 23. Moderate to severe mitral valve stenosis. 24. Severe mitral regurgitation (regurgitant volume ≥ 60 mL/beat, regurgitant fraction ≥50%, and/or effective regurgitant orifice area ≥ 0.40cm2). 25. Primary pulmonary hypertension. 26. Significant restrictive or obstructive pulmonary disease or chronic respiratory condition. 27. Renal failure requiring dialysis. 28. Acute illness, active systemic infection, or sepsis. 29. Significant congenital anomaly or medical problem that, in the opinion of the investigator, would preclude enrollment in this study or compliance with follow-up requirements or would impact the scientific soundness of the clinical trial results. 30. Any woman known to be pregnant or breastfeeding, or any woman of childbearing potential who is not on a reliable form of birth regulation method or abstinence. 31. Current or anticipated participation in any other clinical trial of a drug, device, or biologic during the duration of the study, unless pre-approved by the Sponsor. 32. Presence of intramural thrombus, tumor, or other abnormality that precludes vascular access, catheter introduction, or manipulation. 33. Known drug or alcohol dependency. 34. Life expectancy less than 12 months. 35. Vulnerable subject. |

**Table S2: All Adverse Events**

|  | Adverse events | Total Number of Adverse Events  (n = 85) | Number of Serious Adverse Events  (n = 85) |
| --- | --- | --- | --- |
| Non procedure-related/non-device realted events | **Total events** | **42 (28, 32.9)** | **7 (7, 8.2)** |
|  | COVID-19 infection | 10 (10, 11.8) | 0 (0, 0.0) |
|  | Atrial fibrillation | 4 (3, 3.5) | 3 (3, 3.5) |
|  | Ankle injury | 3 (3, 3.5) | 2 (2, 2.4) |
|  | Groin hematoma **^a,b^** | 3 (3, 3.5) | 0 (0, 0.0) |
|  | Atrial flutter | 2 (2, 2.4) | 0 (0, 0.0) |
|  | Second Degree AVB, Type I **^c^** | 1 (1, 1.2) | 0 (0, 0.0) |
|  | Appendicitis + Appendectomy | 1 (1, 1.2) | 1 (1, 1.2) |
|  | Arm tingling | 1 (1, 1.2) | 0 (0, 0.0) |
|  | Back pain | 1 (1, 1.2) | 0 (0, 0.0) |
|  | Cold Symptoms | 1 (1, 1.2) | 0 (0, 0.0) |
|  | Dysuria | 1 (1, 1.2) | 0 (0, 0.0) |
|  | Fatigue | 1 (1, 1.2) | 0 (0, 0.0) |
|  | Flu Symptoms | 1 (1, 1.2) | 0 (0, 0.0) |
|  | Gastroparesis **^b^** | 1 (1, 1.2) | 0 (0, 0.0) |
|  | Headache | 1 (1, 1.2) | 0 (0, 0.0) |
|  | Heart Failure | 1 (1, 1.2) | 0 (0, 0.0) |
|  | Migraine | 1 (1, 1.2) | 0 (0, 0.0) |
|  | Murmur over the right groin **^b^** | 1 (1, 1.2) | 0 (0, 0.0) |
|  | Palpitations | 1 (1, 1.2) | 0 (0, 0.0) |
|  | Polyp biopsy | 1 (1, 1.2) | 0 (0, 0.0) |
|  | Presyncope | 1 (1, 1.2) | 0 (0, 0.0) |
|  | Sinus Bradycardia | 1 (1, 1.2) | 0 (0, 0.0) |
|  | Thyrotoxicosis | 1 (1, 1.2) | 0 (0, 0.0) |
|  | Urethral pain | 1 (1, 1.2) | 0 (0, 0.0) |
|  | Ureterolithiasis | 1 (1, 1.2) | 1 (1, 1.2) |
| Device and/or procedure-related adverse events | **Total events** | **7 (7, 8.2)** | **1 (1, 1.2)** |
|  | Groin hematoma **^a^** | 5 (5,5.9) | 0 (0, 0.0) |
|  | Atrial flutter | 1 (1, 1.2) | 0 (0, 0.0) |
|  | Diplopia and vertigo **^d^** | 1 (1,1.2) | 1 (1,1.2) |

AVB, atrioventricular block.

Values are reported as incidence number (number of patients, percentage)

**^a^** All groin hematomas were small and did not require intervention.

**^b^** Occurred after remap procedure.

**^c^** Resolved with decreasing beta blockers

**^d^** PULSE2 subject. Symptom remission in two days. Brain CT and MRI evaluation ruled out an embolic event.

**Table S3: Outcomes by Center.**

| Center | Enrollmentt | | Durability † | | One-Year Outcome  (Total Cohort) |
| --- | --- | --- | --- | --- | --- |
|  | **Total Cohort**  **(n=85)** | **PULSE3**  **(n=35)** | **Total Cohort** | **PULSE3** |  |
| Center #1 | **11/85 (13%)** | **0/35 (0%)** | **17/28 (61%)** | **N/A** | **76.5±7.3%** |
| Center #2 | **53/85 (62%)** | **29/35 (83%)** | **153/165 (93%)** | **87/88 (99%)** | **90.9±8.7%** |
| Center #3 | **21/85 (25%)** | **6/35 (17%)** | **44/45 (98%)** | **12/12 (100%)** | **85.7±7.6%** |

**^†^** Data are shown on a per-pulmonary vein basis.

**Table S4. Reconnected Pulmonary Veins During the Remap Procedure.**

| Pulmonary Veins | Total Cohort  (n = 60) | PULSE3  (n = 26) |
| --- | --- | --- |
| Number of reconnected PVs | 24 / 238 (10) | 1 / 100 (1) ^†^ |
| RSPV | 2 / 60 (3) | 0 / 26 (0) |
| RIPV | 6 / 60 (10) | 0 / 26 (0) ^†^ |
| LSPV | 9 / 53 (17) | 1 / 21 (5) |
| LIPV | 5 / 53 (9) | 0 / 21 (0) |
| RMPV | 0 / 0 (0) | 0 / 1 (0) |
| LCPV | 2 / 7 (29) | 0 / 5 (0) |

LCPV, left common pulmonary vein; LIPV, left inferior pulmonary vein; PV, pulmonary vein; RIPV, right inferior pulmonary vein; RMPV, right middle pulmonary vein; RSPV, right superior pulmonary vein.

Values are reported as incidence number (percentage)

^†^ p<0.05 when compared to PULSE1/PULSE2 arm (n=34)

**Figure S1. Diverse Pulmonary Vein Anatomies Treated with the Single-shot Pulsed Field Ablation Catheter.**

**
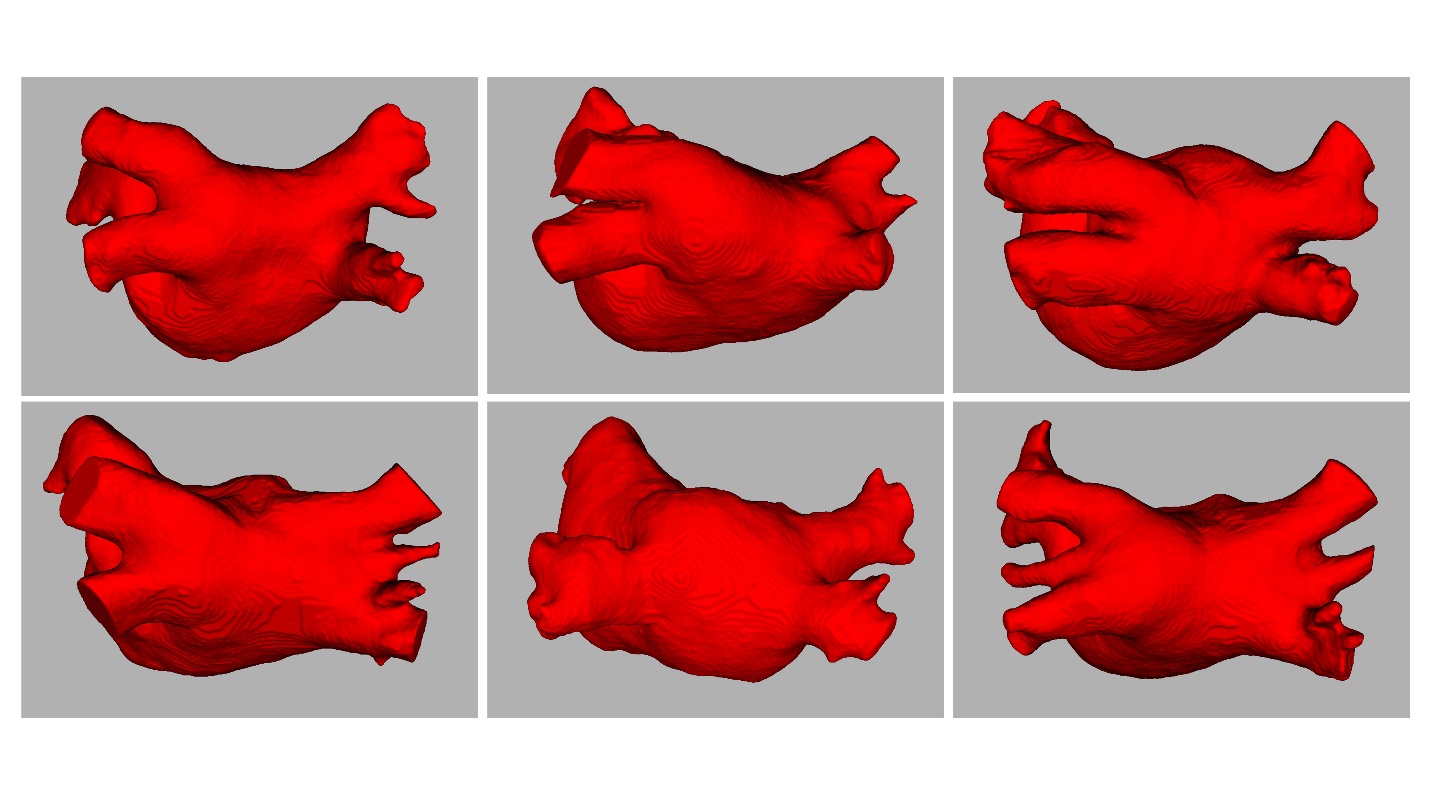
**

**Figure S1. Diverse Pulmonary Vein Anatomies Treated with the Single-shot Pulsed Field Ablation Catheter.** Shown are 3D CT images of six different PV anatomies that were treated with the single-shot PFA catheter.

**Figure S2: Durability of PV Isolation of all Three Pulsed Field Waveforms**


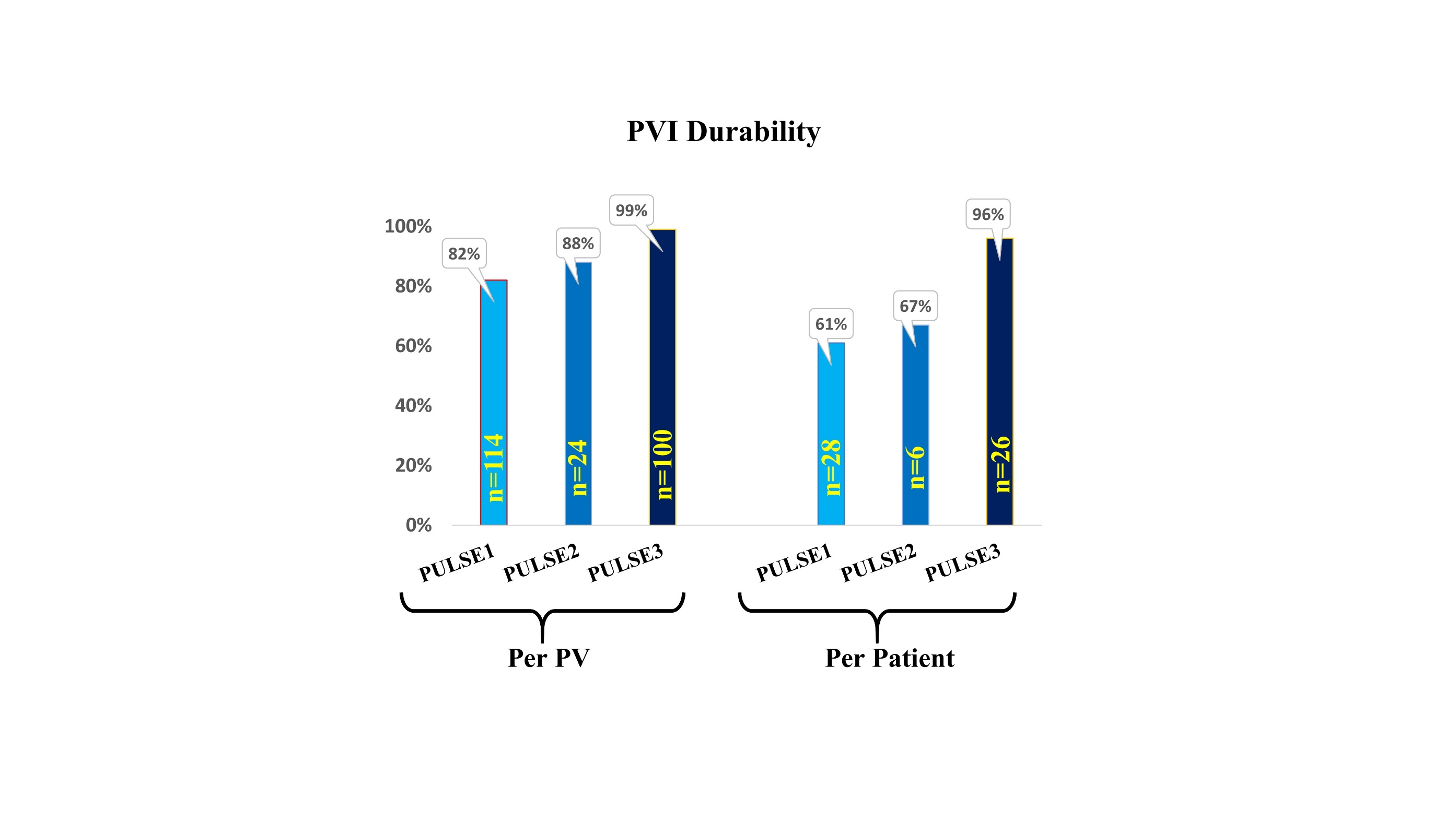


**Figure S2: Durability of PV Isolation of all Three Pulsed Field Waveforms.** Shown are the durability of PV isolation for each of the 3 pulsed field waveforms, represented both on a per-vein basis (left) and on a per-patient basis (right). With the most optimized PULSE3 waveform, durability on a per-vein and per-patient basis was optimal at 99% and 96%, respectively. PV, pulmonary vein; PVI; pulmonary vein isolation.

**Figure S3: PULSE3 Patient with a PV Reconnection: Electroanatomical Mapping.**

**
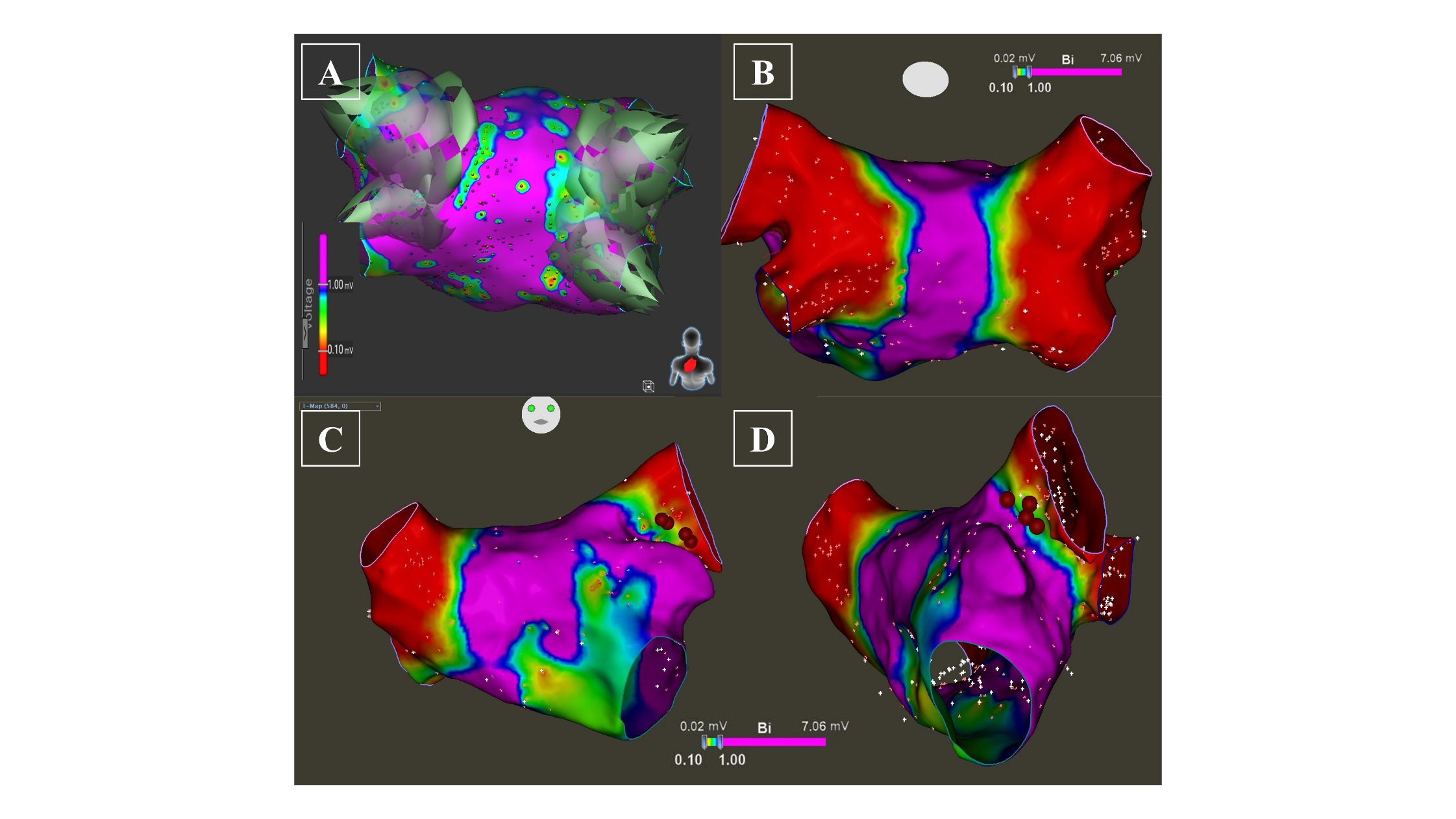
**

**Figure S3: PULSE3 Patient with a PV Reconnection: Electroanatomical Mapping**. Shown are the locations of the pulsed field applications, represented as green shadows (**A**) during the index ablation procedure, and during the remapping procedure, the electroanatomical bipolar voltage amplitude maps in posterior view (**B**), anterior view (**C**) and left-anterior oblique view (**D**). The radiofrequency ablation tags (red dots) to ablate the PV reconnections during the redo procedure are shown in (**C**) and (**D**). The color range is 0.1 (red) to 1.0 mV (purple).

**Figure S4: PULSE3 Patient with a PV Reconnection: Mapping & Ablation.**

**
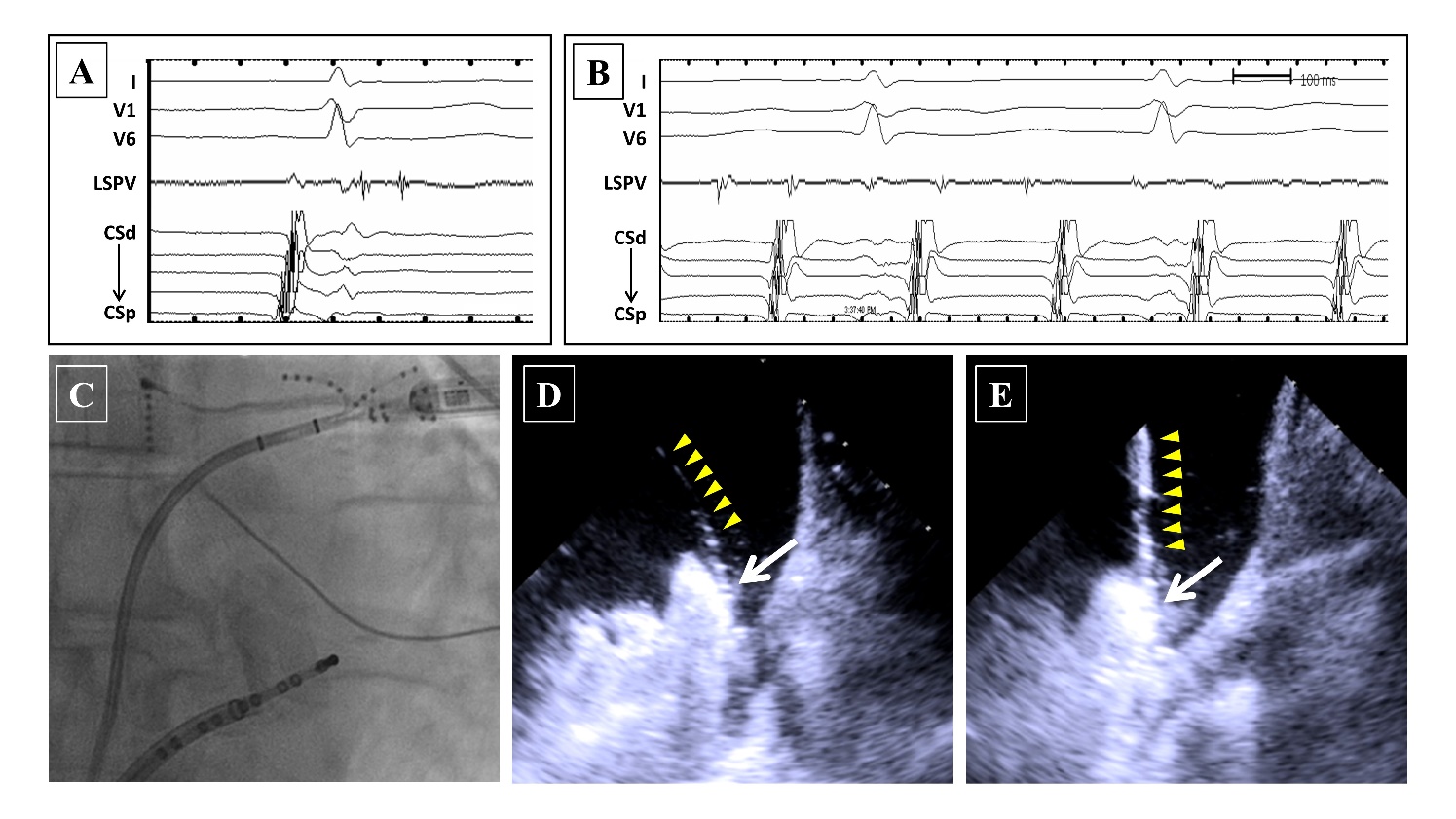
Figure S4: PULSE3 Patient with a PV Reconnection: Mapping & Ablation**. (**A**) In the anterior-ridge aspect of the left superior PV, there was a fragmented electrogram with delayed components extending even beyond the end of the QRS complex. (**B**) During the procedure, spontaneous firing from this PV resulting in salvos of transient organized-looking atrial tachycardia. (**C**) The location of the mapping catheter in the left superior PV is shown on fluoroscopy. (**D-E**) The location of the radiofrequency ablation catheter (yellow arrowheads) at the ridge is shown in these two intracardiac ultrasound images (white arrows denote the ablation tip).
