# Supplementary figures and images for: First-in-human clinical series of a novel conformable large-lattice pulsed field ablation catheter for pulmonary vein isolation
Source: Europace. 2024 Apr 8;26(4):euae090. doi: 10.1093/europace/euae090 (PMC11057205; doi:10.1093/europace/euae090)

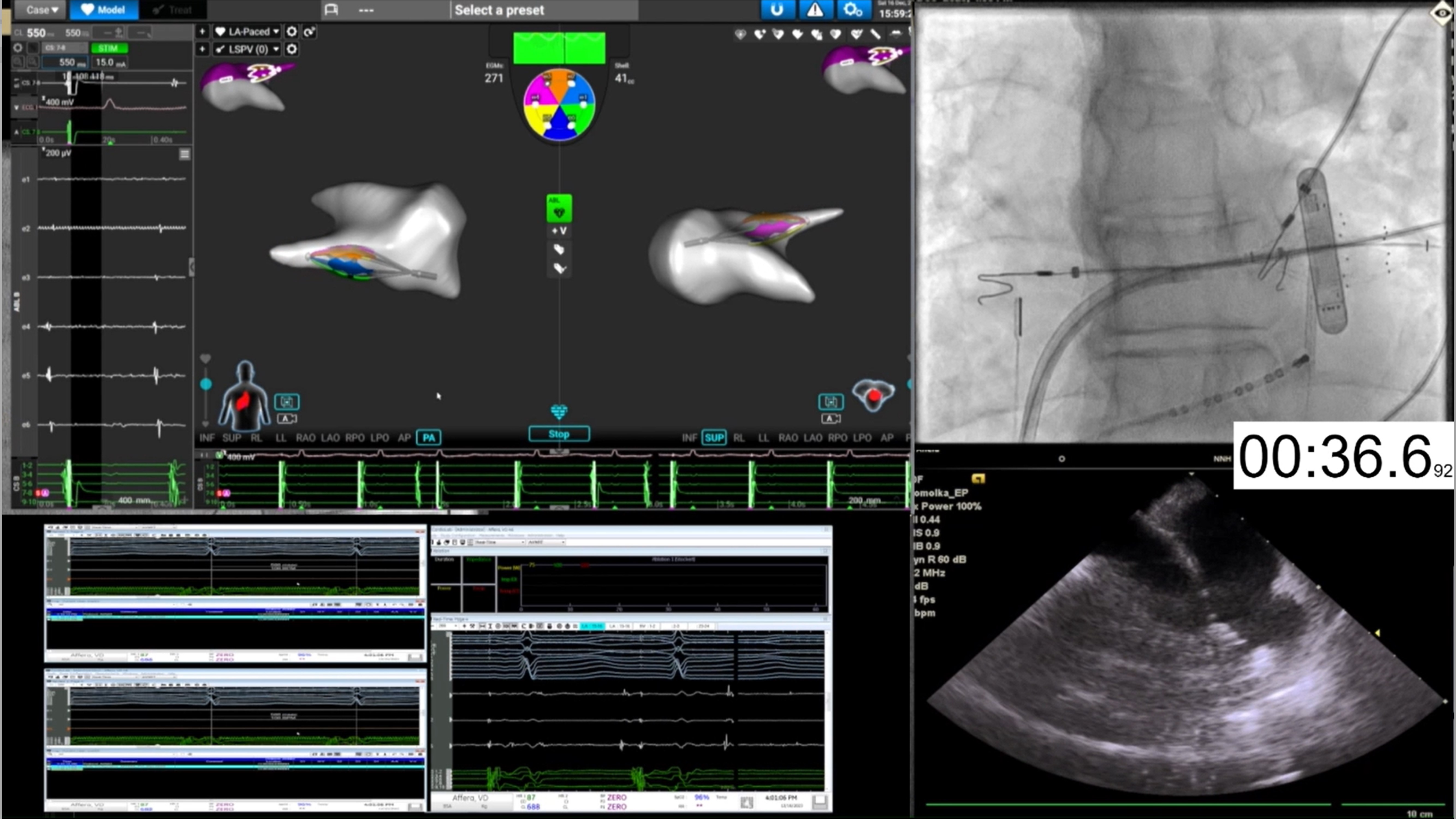

Supplement: euae090_Supplementary_Data [file euae090_supplementary_data.zip › Video 1 Still Image.JPG]

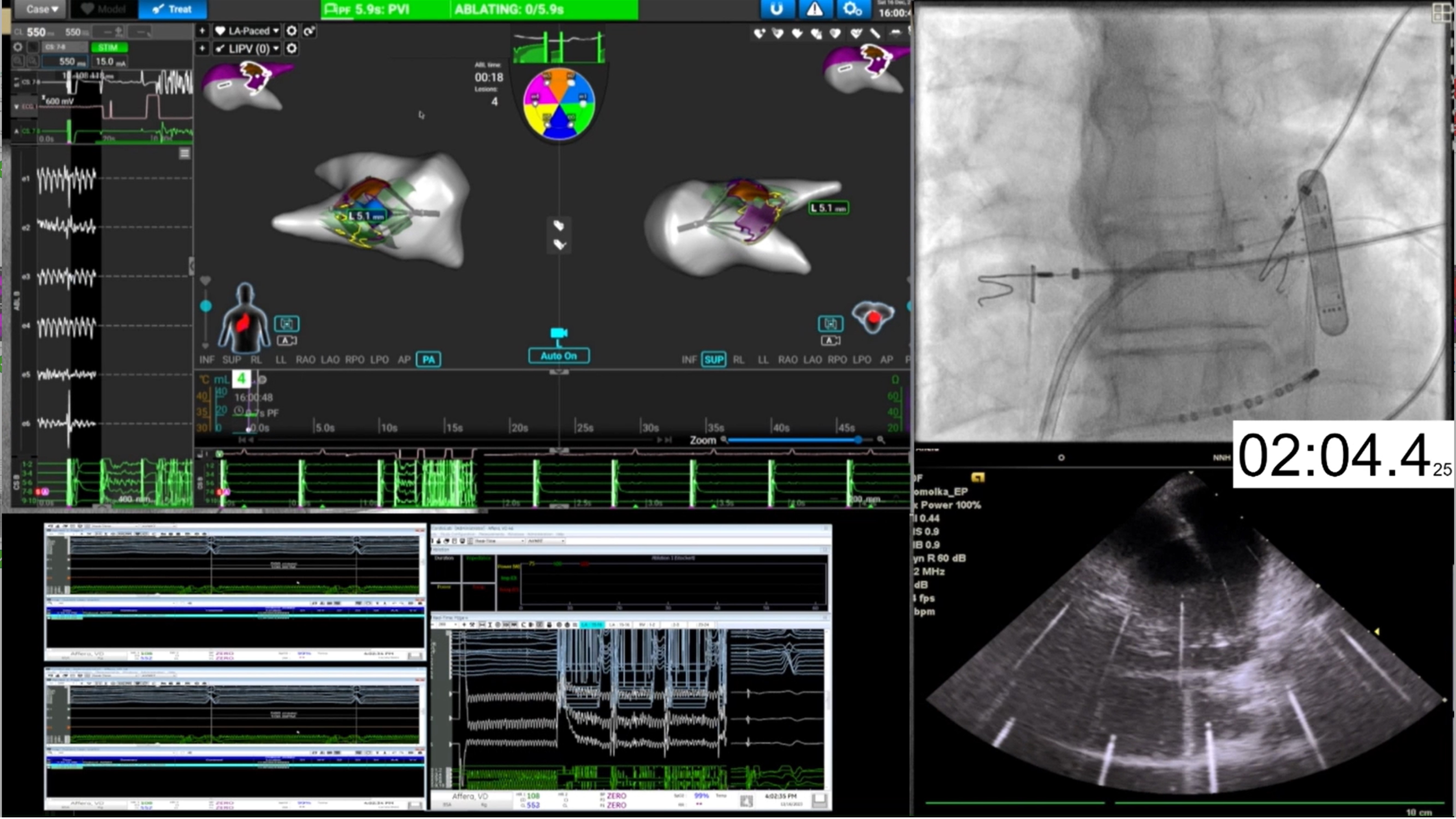

Supplement: euae090_Supplementary_Data [file euae090_supplementary_data.zip › Video 2 Still Image.JPG]
